# A method for rapid and homogenous initiation of post-harvest physiological deterioration in cassava storage roots identifies Indonesian cultivars with improved shelf-life performance

**DOI:** 10.1186/s13007-022-00977-w

**Published:** 2023-01-18

**Authors:** Ima M. Zainuddin, Brieuc Lecart, Enny Sudarmonowati, Hervé Vanderschuren

**Affiliations:** 1 Department of Biology, Plant Biotechnology, Eidgenössische Technische Hochschule (ETH) Zurich, Universitätstrasse 2, 8092 Zurich, Switzerland; 2 Department of Biosystems, KU Leuven, Willem de Croylaan 42, Box 2455, 3001 Louvain, Belgium; 3 Institut Teknologi Bandung (ITB), Jl. Ganesha 10, Bandung, 40132 Indonesia; 4 Plant Genetics, Gembloux Agro-Bio Tech, University of Liège, Passage Des Déportés 2, 5030 Gembloux, Belgium; 5 Research Center for Genetics Engineering, National Research and Innovation Agency (BRIN), Jl. Raya Bogor Km. 46, Cibinong, 16911 Indonesia

**Keywords:** PPD assessment, Cassava germplasm

## Abstract

Cassava is the most cultivated and consumed root crop in the world. One of the major constraints to the cassava value chain is the short shelf life of cassava storage roots which is primarily due to the so-called post-harvest physiological deterioration (PPD). The identification of natural sources of PPD tolerance represents a key approach to mitigating PPD losses by generating farmer- and industry-preferred cassava cultivars with prolonged shelf life. In the present study, a PPD assessment method was developed to screen for PPD tolerance in the cassava germplasm. The proposed PPD assessment method displayed a reduced rate of microbial infection and allowed a rapid and homogenous development of typical PPD symptoms in the cassava storage roots. We successfully used the PPD assessment method in combination with an image-based PPD scoring method to identify and characterize PPD tolerance in 28 cassava cultivars from the Indonesian cassava germplasm. Our analysis showed a significant and positive correlation between PPD score and dry matter content (r = 0.589–0.664, p-value < 0.001). Analysis of additional root parameters showed a significant and positive correlation between PPD scores at 2 days post-harvest (dph) and root length (r = 0.388, p-value < 0.05). Our analysis identified at least 4 cultivars displaying a significantly delayed onset of PPD symptoms as compared to the other selected cultivars. The availability of cassava cultivars contrasting for tolerance to PPD will be particularly instrumental to understanding the molecular mechanisms associated with delayed PPD in cassava roots.

**Supplementary Information:**

The online version contains supplementary material available at 10.1186/s13007-022-00977-w.

## Introduction

Also called yuca (Spanish), manioc (French), mandioc (Portuguese), cassave (Dutch), and maniok (German), cassava (*Manihot esculenta* Crantz) is widely cultivated in tropical countries located in the equatorial belt, between 30° North and 30° South of the Equator [14]. Cassava is the fourth most important staple crop in the world after rice, wheat, and maize [13, 14]. It provides staple food to nearly a billion people in over 100 countries and it is recognized as the cheapest source of starch used in more than 300 industrial products [13].

Cassava production in Asia represents 27% of the global production and Indonesia is the fifth cassava producer after Nigeria, the Republic Democratic of Congo, Thailand, and Ghana [15]. In Indonesia, cassava also ranks as the fourth most important food crop after rice, maize, and soybean. Over the last decade, cassava productivity in Indonesia has steadily increased. Cassava production in Indonesia is spread throughout the provinces with the highest production in Lampung and Java provinces [27]. Indonesian cassava production is mainly used for the local food industry (51.9%), other industries and exports (39.4%), as well as direct consumption (4.3%) [1].

Despite good agronomic performance, cassava production remains constrained by the high perishability of its storage roots [25, 43]. The short shelf life of cassava root is primarily due to the so-called post-harvest physiological deterioration (PPD), which affects most cassava cultivars used by the starch industry and small-scale farmers [33, 49]. Due to PPD, cassava roots cannot be kept in satisfactory conditions for more than a few days after harvest [3]. The bulkiness and perishability of cassava roots hamper the global trade of fresh cassava, which has long been recognized as a market with high potential for expansion provided that cost-effective methods to delay PPD can be implemented [14]. It has been estimated that extending the storability of cassava to 45 days would lead to an increase in annual benefits of approximately US $ 35 million in Thailand [16, 42]. An *ex-ante* impact study also indicated that the development of cassava varieties with delayed PPD would increase cassava benefits by US $ 2.9 billion, $ 855 million, FAO/IFAD and $ 280 million in respectively Nigeria, Ghana, and Uganda [32].

Genetic improvement of cassava is a long and tedious process [6, 9] but it could represent a cost-effective approach to address the issue of PPD, in particular, if delayed PPD trait with high heritability could be identified and introgressed with molecular markers [23, 32]. Sources of PPD tolerance have been identified in mutated cultivars (i.e. mutant line 2G15-1 and amylose-free starch mutant line AM 206-5), interspecific hybrid between *Manihot esculenta* and its wild relative *Manihot walkerae* as well as yellow-rooted cassava genotypes with high carotene content [7, 8, 22, 37]. Genetically, the high heterozygosity of cassava renders the introgression of the trait of interest complicated as properties from the recurrent parental line need to be recovered through tedious backcross schemes [8]. Moreover, recent studies have revealed the significance of G × E interaction for the PPD tolerance trait in selected cultivars [24, 38]. Therefore, identification and characterization of additional sources of PPD tolerance in cultivars combining other important specificities such as good agronomic performance in local environments and preference by farmers and industry could help generate cassava cultivars with delayed PPD traits using simpler and shorter breeding schemes.

Assessment of the shelf-life performance is also difficult due to the large experimental errors associated with the available PPD initiation and scoring protocols [16, 22], reviewed in [49]. Most PPD assessment methods rely on the initiation of PPD by removing the proximal and distal ends of the cassava storage roots (Wheatley and Gomez, 1985). A PVC film is often used to cover the distal end of the 15 cm long root and to prevent moisture loss. Gradients of increasing PPD symptoms from the proximal to distal ends of the storage root can then be observed [34]. This method has been extensively used in cassava PPD studies [10, 20, 35, 37, 39, 46, 47, 50]. The protocol usually allows initiation of PPD from the proximal end of the root but variation in PPD symptoms can be observed within the storage roots from the same genotype [16]. The visual scoring of PPD symptoms commonly used with the standard PPD assessment methods represents another source of variation. Therefore, image-based scoring systems have also been implemented to reduce the experimental errors associated with visual scoring [41, 47, 50].

To generate samples with rapid and homogenous PPD response for transcriptomics analysis, Reilly and colleagues (2004; 2007) performed two longitudinal V-shaped cuts on the cassava roots. We previously used a root slice method which allowed a rapid and homogenous onset of PPD symptoms for proteomics studies [26, 41]. Despite the increased homogeneity in PPD symptoms, the use of longitudinal V-shaped cuts or slicing methods to initiate PPD can be problematic as they represent extended entry points for pathogens. Under high humidity and temperature conditions, such methods can be particularly prone to the development of pathogenic microorganisms growing on cassava storage root tissues and producing symptoms that are barely distinguishable from PPD symptoms. Microbial infections of storage roots lead to the so-called secondary deterioration as it often takes place when the roots have already undergone physiological deterioration [3]. Secondary deterioration is particularly important when roots experience severe damage at harvest [33, 36]. The use of roots with limited damage during harvest often reduces the incidence of microbial infection during the early phases of storage. In some cultivars, the use of storage roots with minimal damage can also significantly delay the initiation of PPD [22]. The late development of PPD sometimes makes the distinction between physiological deterioration and microbial rotting difficult.

Here we report the development of a PPD tolerance assessment method relying on a protocol initiating homogenous PPD symptoms. We used image-based analysis to test the reliability and robustness of the protocol. We subsequently used the protocol to assess the PPD tolerance trait in selected accessions from the Indonesian cassava germplasm collection to identify cultivars with reduced PPD onset after harvest.

## Materials

### Plant and root materials

Twenty-eight cassava cultivars cultivated in several cassava-producing regions in Indonesia (Additional file 1: Data S1) were chosen from the core cassava germplasm collection maintained at the National Research and Innovation Agency (BRIN), Indonesia. The cultivars were selected based on their agronomic performance under local field conditions as well as their preference by farmers and industries. The selected cultivars, which were multiplied by stem cuttings, were planted and harvested at RC Biotech LIPI (BRIN)’s field in 2012 and 2014. Cassava plants were harvested at 8–9 months after planting in 2012 and at 10–12 months after planting in 2014. The harvested roots were weighed and measured for their diameter and length prior to using them for the PPD assay.

## Methods

### PPD assessment

#### Standard PPD assessment method

**Fig. 1:**
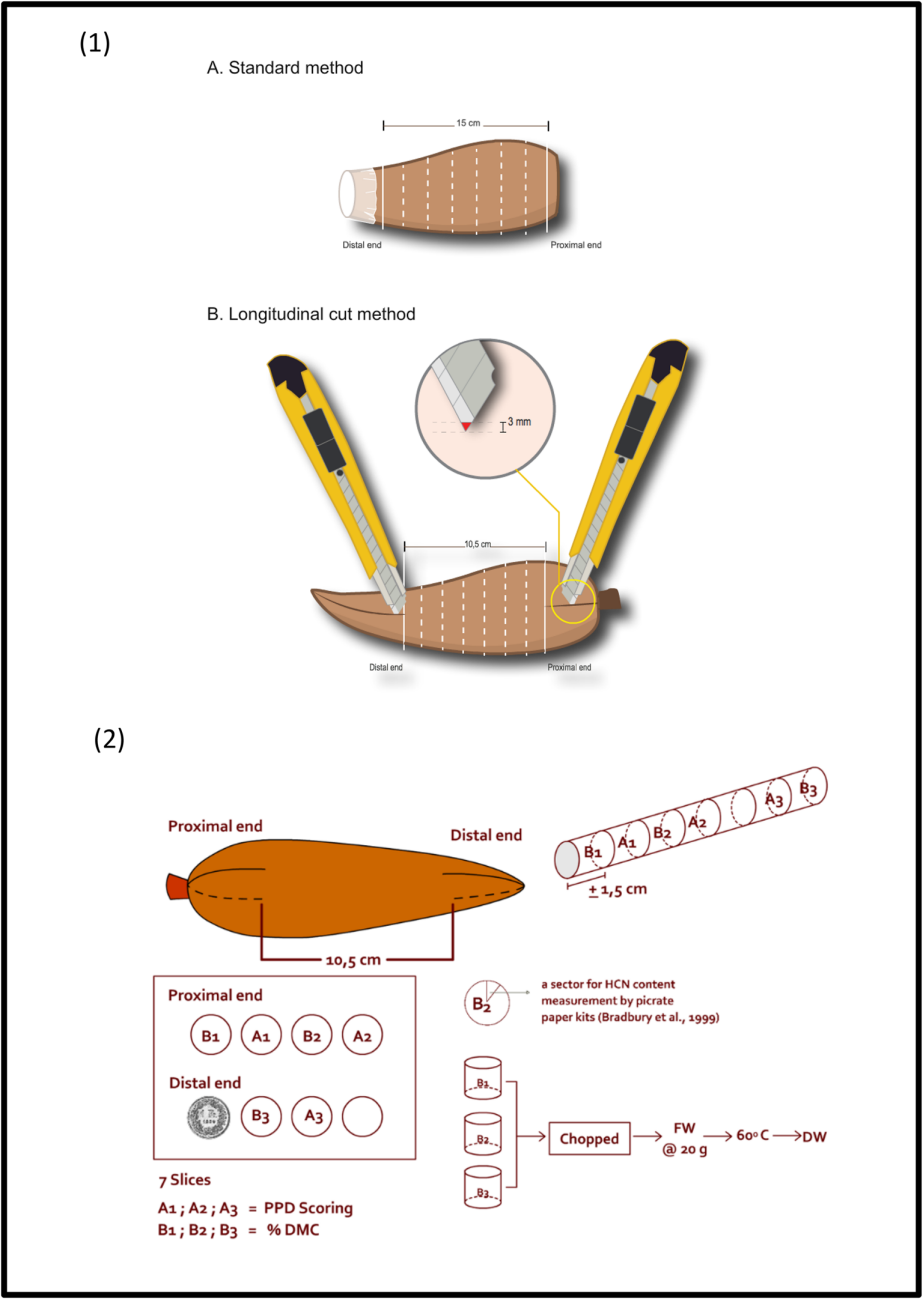
(1) Illustration of PPD initiation by using standard and longitudinal cut methods; (2) Illustration of sampling used for DMC and HCN content measurements. **A** detailed description of the methods is provided in Material and Methods as well as Supplementary Methods

The PPD assessment method developed by Wheatley and colleagues (1985) was used as the standard method. The proximal and distal ends of the roots were cut to leave a remaining uncut root section that was at least 15 cm long. The used knife was dipped in ethanol 96% and distilled water before making the cut. The distal end was then covered with PVC film in order to maintain moisture content at the distal end and to inhibit the PPD occurrence from this section (Fig. 1).

#### Longitudinal cut method

The longitudinal method consisted of a partial shallow cut of the cassava roots as illustrated (Fig. 1) and described in more detail in Additional file 3. The 3 mm-deep cuts were performed with a paper cutter at both ends of the roots, leaving the central part of the root (10.5 cm long) undamaged. The undamaged central part was sliced into 7 transversal slices (ca. 1.5 cm thickness) to analyze PPD symptoms. The PPD symptoms were evaluated at 0, 2, 4, 7 and 14 days post-harvest (dph).

The storage place for all treated roots was designed to be protected from direct sun, rain, and rodents, but exposed to air. The average temperature and relative humidity in the storage place during the PPD assessment are provided in Additional file 4: Table S1.

### Dry matter content (DMC) and HCN content measurement

DMC and HCN contents were evaluated at 0 dph. Three root slices per root (ca. 1.5 cm thickness) were pooled (Fig. 1) and used to determine the DMC. Data were collected from four roots per cultivar. The dry weight value was obtained by drying 20 g of the pooled roots in an oven at 60 °C for 3 days. DMC was expressed as the percentage of dry weight relative to fresh weight. HCN measurements were performed on root slices from the middle section of the root (Fig. 1). Total cyanide content was determined with picrate paper kits following an established protocol [4].

### Matlab-based image processing tool

Digital images of 7 root slices (ca. 1.5 cm thickness) per root were recorded by Canon EOS 500D. Images of proximal, central and distal slices (Fig. 1) were used for PPD score analysis using a previously established image-based PPD scoring procedure [41]. The MATLAB algorithm converted the image from RGB (red, green, blue) color to grayscale and allowed the user to select an area of interest. A histogram of the monochrome image based on normalized pixel frequencies was generated from the algorithm. PPD score was defined from the following formula:

$${\text{PPD}}\,{\text{score}} = {{({\text{Quantile}}\,97.5\% - {\text{Quantile}}\,2.5\% )} \mathord{\left/ {\vphantom {{({\text{Quantile}}\,97.5\% - {\text{Quantile}}\,2.5\% )} {{\text{Quantile}}\,97.5}}} \right. \kern-0pt} {{\text{Quantile}}\,97.5}}\%$$

PPD score was calculated by averaging the PPD scores from the proximal, central, and distal slices.

### Statistics analysis

One-way analysis of variance (ANOVA) F-test was used to analyze the equality of the two tested methods. The F-test was calculated by using the values of the standard deviation of PPD scores per cultivar per method at 4 and 7 dph. The test was performed with R 3.1 software with the following model:

$${\text{sdPPD}} \sim {\text{mt }} * {\text{accs + tp}}$$

where: sdPPD, a standard deviation of PPD scores per assessed cultivar at 4 and 7 dph; mt, methods (standard and longitudinal cut); accs, cultivars; tp, time points. The model was applied to 4 and 7 dph data sets based on the normal distribution of the PPD scores.

ANOVA and Honest Significant Difference (HSD) post hoc test at 4 and 7 dph with factor coding method was used to analyze the significance of microbial contamination to the two tested methods and cultivars. The PPD scores of all evaluated cultivars at 4 and 7 dph obtained with the longitudinal cut method were subsequently subjected to ANOVA and Least Significant Difference (LSD) post hoc test at a 5% significance level. ANOVA test and Shapiro–Wilk to test the normality of the data were performed by XLSTAT.

### Classification of the assessed cultivars

The twenty-eight cassava cultivars were compared based on the average PPD score at 0, 2, 4, and 7 dph (average PPD score) and k-means clustering provided by XLSTAT software. The patterns of their PPD development were grouped into seven distinct clusters. Clusters 1–2 are classified as delayed PPD, clusters 3–4 as intermediate PPD, and clusters 5–7 as early PPD.

### Principal component analysis (PCA)

PCA was performed on a dataset comprising root diameter, microbial contamination, cyanide content and DMC measurement for the twenty-eight cassava cultivars from 2012. Analysis was conducted in R [28] and the following packages: FactoMineR, factoextra, Rcmdr, and ggsci.

## Results

### The longitudinal cut method allows robust assessment of PPD

A preliminary test in 2011 using the standard PPD assessment method was performed with AdiraI, AdiraIV, MentegaI, MentegaII, and Roti cassava cultivars under local conditions. PPD only initiated and developed from the proximal end but we observed that a large fraction of cassava roots was undergoing rapid microbial contamination after the removal of both root ends (Additional file 4: Table S2). In order to reduce the level of microbial contamination, we also implemented an alternative method by replacing the removal of proximal and distal ends with two longitudinal cuts on both ends of the storage root (Fig. 1; Additional file 3) and compared the longitudinal cut method to the standard PPD assessment method. We hypothesized that shallow longitudinal cuts would allow homogenous entry of oxygen to the parenchymatous root tissues and limit the level of microbial contamination. The PPD symptoms were evaluated at 0, 2, 4, 7, and 14 days post-harvest (dph).

**Fig. 2:**
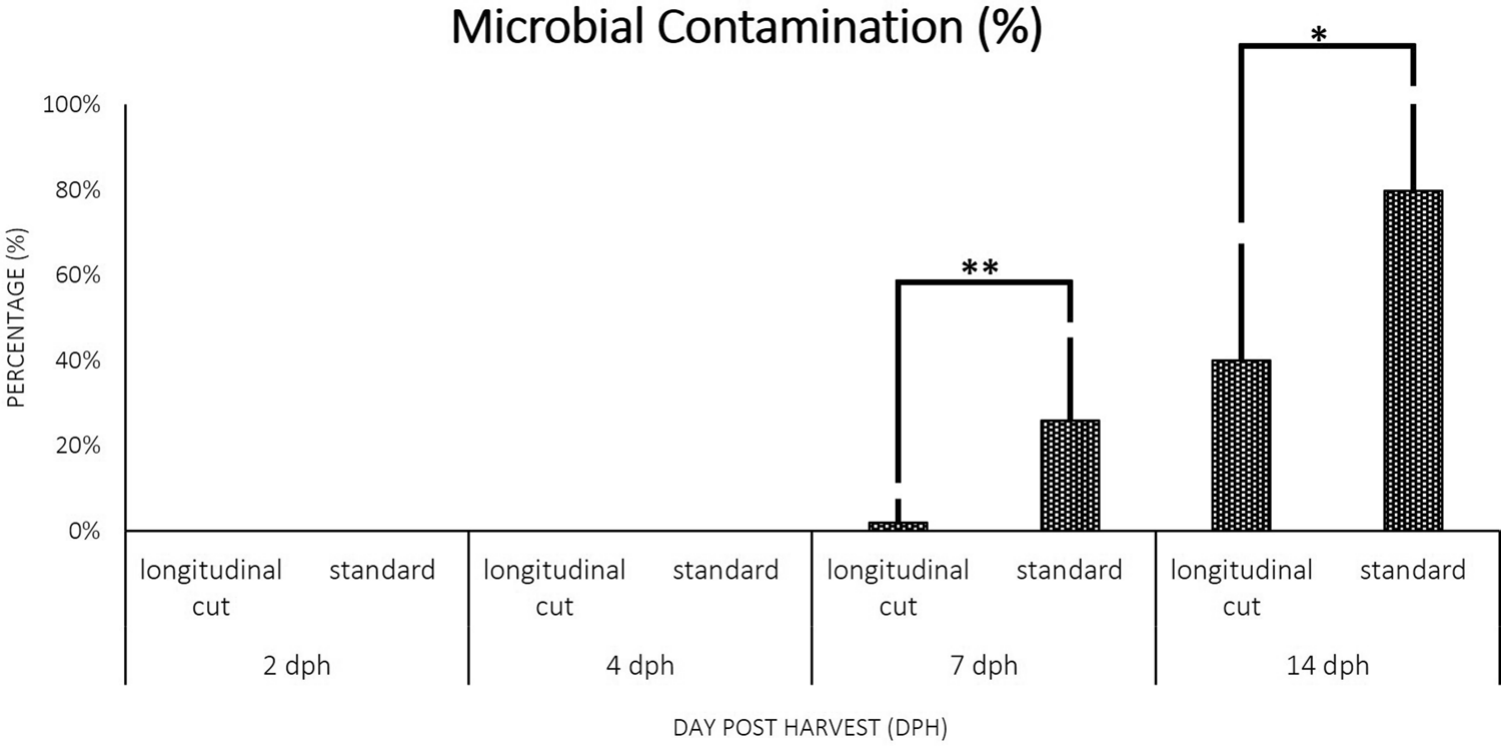
The percentage of microbial contamination in nine cassava cultivars was assessed by longitudinal cut and standard methods at 7 and 14 dph. Asterisk (* and **) shows significance level at 5% and 1% respectively, determined by student t-test between the two methods at the same time point

Both standard and longitudinal cut methods were initially assessed using a selection of 9 cultivars. Roots to be used for PPD assessment with each method were randomly chosen. Visual observations made at 7 dph and 14 dph confirmed that the standard PPD assessment method was prone to microbial contamination. The proximal ends appeared to show higher microbial contamination rates with the standard assessment method. Using the standard PPD assessment method, seven out of nine cultivars displayed microbial deterioration (17–60%) at 7 dph and all nine cultivars displayed microbial contamination (25–100%) at 14 dph (Fig. 2; Additional file 4: Table S3). Noticeably the high percentage of contamination (up to 100%) occurring in several cassava cultivars at certain time points considerably reduced the number of assessed roots that could be used for PPD scoring. Image-based PPD scoring was applied to all roots treated with the PPD assessment methods. Because the matlab algorithm converts the image from RGB color to grayscale, colored sections of cassava root slices caused by microbial infection, such as dark black color due to fungal and yellowish color due to bacterial contamination (Additional file 2: Figure S1), could not be distinguished from typical PPD symptoms. Therefore the contaminated roots were not used for PPD scoring as their strong coloration associated with microbial or secondary deterioration would interfere with scoring the level of PPD in those roots.

Using the longitudinal cut method, the percentage of microbial infection was only detected in one out of nine cassava cultivars at 7 dph (Additional file 4: Table S3). Although microbial infection progressed for both methods at 14 dph, the microbial infection rate remained significantly lower at 14 dph with the longitudinal cut method as compared to the standard method (Fig. 2). Noticeably, two cultivars remained without microbial contamination at 14 dph with the longitudinal cut method. The statistical analysis with ANOVA and HSD post hoc test on microbial contamination versus methods, cultivars and time points at 7 and 14 dph revealed that the longitudinal cut method had a microbial contamination rate significantly lower as compared to the standard method (p-value < 0,05; Additional file 4: Table S4).

**Fig. 3:**
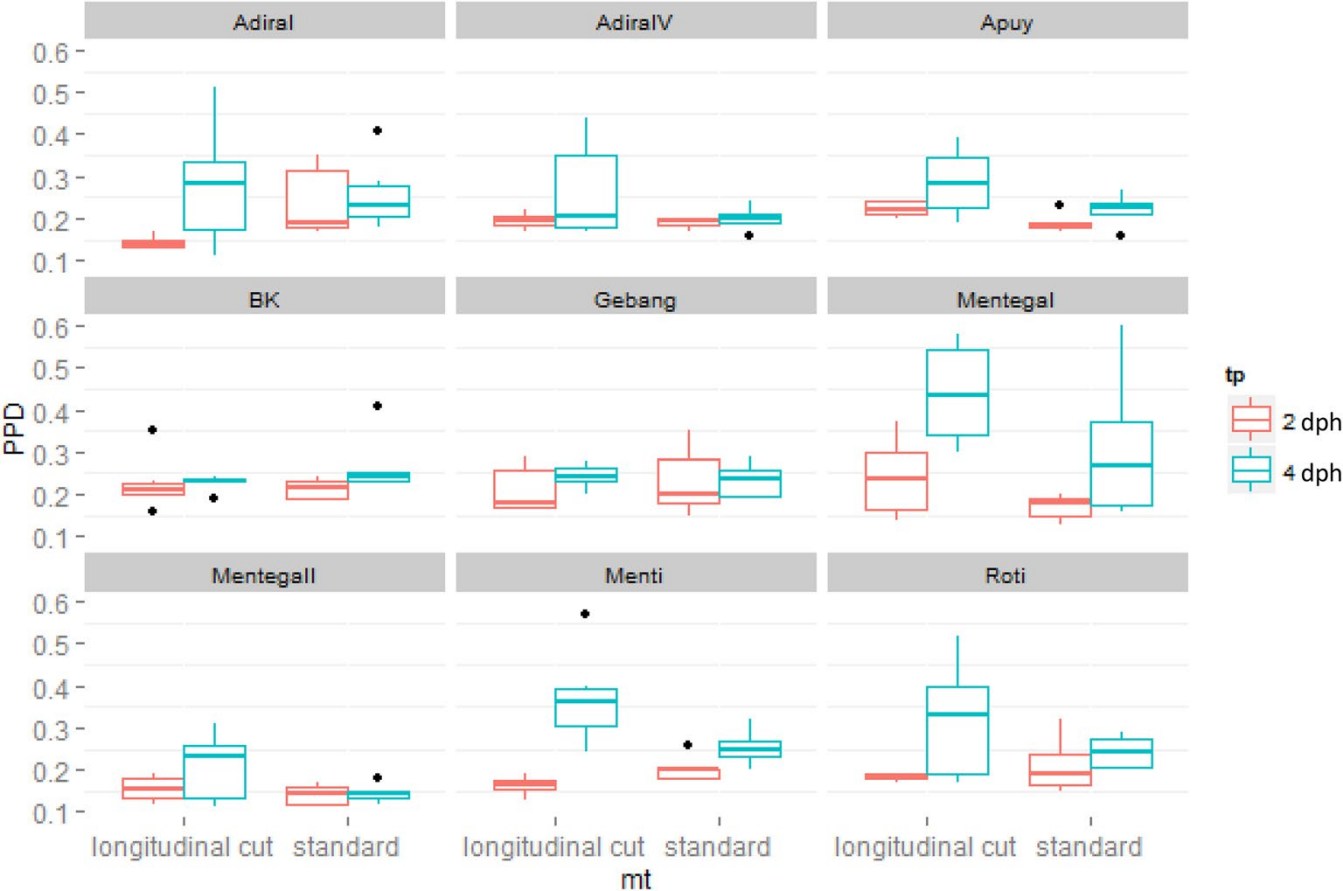
PPD development of nine cassava cultivars assessed by longitudinal cut and standard methods at 2 and 4 dph

Importantly, the longitudinal cut method resulted in a more rapid initiation of PPD as illustrated by the higher PPD scores at 4 dph (Fig. 3). In order to assess the variation in PPD symptom development for both methods, we analyzed the experimental standard deviation by performing an ANOVA F-test on the PPD scores at 4 and 7 dph. Both time points were chosen because of the normal distribution of the PPD scores. An F-test analysis showed that there is no significant difference between the two methods across the cassava cultivars under evaluation (Table 1), indicating that both methods can trigger a homogenous initiation of PPD in cassava storage roots.

**Table 1:** ANOVA F Test to analyze the equality of two evaluated methods.

sdPPD ~ mt * ct + tp						
	Df	Sum_of_Sq	RSS	AIC	F_value	Pr(> F)
< none >			0.023614	− 225.86		
tp	1	0.0022336	0.025848	− 224.61	1.608	0.2219 (ns)
mt:cv	8	0.0080067	0.031621	− 231.35	0.7205	0.6717 (ns)

The F-test was calculated by using the values of standard deviation of PPD scores per cultivar per method at 4 and 7 dph

*Ns* not significant, *tp* time points, *mt *methods, *cv* cultivars

Because the longitudinal cut method outperformed the standard method for microbial contamination, it was selected as a method of choice to further investigate differences in PPD onset in the cassava germplasm.

### Identification of cassava cultivars with contrasting response to PPD

Using the longitudinal cut method, nineteen additional Indonesian cassava cultivars were screened for their response to PPD. PPD scores at 2, 4 and 7 dph were used to characterize their shelf-life performance (Table 2). We compared all cassava cultivars and classified them based on the average PPD score (Table 2). We also performed k-means clustering to distinguish different patterns of PPD onset. Using PPD scores at 2, 4 and 7 dph, PPD responses of the selected cassava could be grouped into seven distinct clusters (Additional file 2: Figure S2). Clusters 1 and 2 correspond to the PPD development patterns of cultivars with lower average PPD score (average PPD scores < 0.29) as delayed PPD while clusters 5 and 7 correspond to cultivars with early onset of PPD (average PPD scores ≥ 0.29). Manggu, MentegaII, Kristal Merah, Valenca, Baros Kencana, Gebang, AdiraIV, Apuy, Roti, and Darul Hidayah are all grouped in clusters 1 and 2 with average PPD scores < 0.29, so that classified as delayed PPD.

**Table 2:** PPD scores of 28 Indonesian cassava cultivars

No.	Cultivars	Level of greyness (PPD Score)				%contaminated roots at 7 dph %	Average PPD Score	PPD status	Cluster
		0 or 1 dph	2 dph	4 dph	7 dph				
1	Manggu	0.15 ± 0.02	0.15± 0.06	0.18 ± 0.06	0.21 ± 0.07	11	0.17	delayed	1
2	MentegaII	0.13 ± 0.02	0.16 ± 0.03	0.21 ± 0.08	0.22 ± 0.07	0	0.18	delayed	1
3	Kristal Merah	0.14 ± 0.01	0.18 ± 0.09	0.25 ± 0.1	0.27 ± 0.13	0	0.21	delayed	1
4	Valenca	0.12 ± 0.02	0.21 ± 0.1	0.28 ± 0.09	0.24 ± 0.06	0	0.21	delayed	1
5	Baros Kencana	0.2 ± 0.02	0.23 ± 0.06	0.23 ± 0.02	0.22 ± 0.04	0	0.22	delayed	2
6	Gebang	0.19 ± 0.03	0.21 ± 0.06	0.24 ± 0.03	0.28 ± 0.05	0	0.23	delayed	2
7	AdiraIV	0.18 ± 0.01	0.2 ± 0.02	0.27 ± 0.12	0.28 ± 0.06	0	0.23	delayed	2
8	Apuy	0.18 ± 0.02	0.22 ± 0.02	0.29 ± 0.08	0.27 ± 0.07	0	0.24	delayed	2
9	Roti	0.17 ± 0.01	0.18 ± 0.01	0.33 ± 0.15	0.28 ± 0.09	0	0.24	delayed	2
10	Darul Hidayah	0.19 ± 0.03	0.26 ± 0.09	0.28 ± 0.04	0.31 ± 0.07	17	0.26	delayed	2
11	Lokal Nguneng	0.13 ± 0.02	0.24 ± 0.19	0.33 ± 0.08	0.18 ± 0.05	50	0.22	intermediate	3
12	Randu	0.11 ± 0.01	0.2 ± 0.1	0.35 ± 0.15	0.3 ± 0.14	33	0.24	intermediate	3
13	Lelen	0.14 ± 0.02	0.26 ± 0.1	0.41 ± 0.13	0.2 ± 0.11	50	0.25	intermediate	3
14	Menti	0.16 ± 0.01	0.16 ± 0.02	0.37 ± 0.11	0.33 ± 0.11	17	0.26	intermediate	4
15	Ubi Kuning	0.15 ± 0.02	0.21 ± 0.07	0.34 ± 0.08	0.34 ± 0.12	0	0.26	intermediate	4
16	AdiraI	0.13 ± 0.03	0.14 ± 0.01	0.28 ± 0.15	0.5 ± 0.17	0	0.26	intermediate	4
17	Malang II	0.12 ± 0	0.25 ± 0.12	0.32 ± 0.09	0.4 ± 0.09	40	0.27	intermediate	4
18	Rengganis	0.14 ± 0.02	0.27 ± 0.13	0.36 ± 0.21	0.42 ± 0.11	20	0.30	early	5
19	Kristal Putih	0.14 ± 0.01	0.34 ± 0.04	0.38 ± 0.16	0.4 ± 0.17	0	0.32	early	5
20	Gempol	0.17 ± 0.01	0.33 ± 0.11	0.41 ± 0.13	0.36 ± 0.1	0	0.32	early	5
21	Malang VI	0.17 ± 0.01	0.4 ± 0.12	0.4 ± 0.1	0.37 ± 0.06	33	0.34	early	5
22	Sentul	0.17 ± 0.03	0.33 ± 0.1	0.47 ± 0.14	0.39 ± 0.1	33	0.34	early	5
23	MentegaI	0.14 ± 0.02	0.24 ± 0.09	0.44 ± 0.12	0.32 ± 0.19	0	0.29	early	6
24	Baturaja	0.16 ± 0.05	0.24 ± 0.13	0.4 ± 0.07	0.4 ± 0.11	10	0.30	early	6
25	BIC302	0.14 ± 0.01	0.27 ± 0.13	0.46 ± 0.06	0.46 ± 0.06	0	0.33	early	7
26	Tali	0.15 ± 0.02	0.3 ± 0.07	0.52 ± 0.11	0.5 ± 0.09	33	0.37	early	7
27	Ubi Putih	0.15 ± 0.01	0.4 ± 0.07	0.49 ± 0.07	0.45 ± 0.13	0	0.37	early	7
28	Vandemir	0.14 ± 0.01	0.47 ± 0.11	0.49 ± 0.14	0.55 ± 0.05	11	0.41	early	7

All evaluated cultivars were ranked based on the average PPD score. The pattern of PPD development within 7 dph was divided into seven groups (Additional file 2: Figure S2). The number of biological replicates per cultivar per time point: Additional file 1: Data S4

Clusters 3–4 are considered as intermediate because their average PPD scores are noticeably low (0.22 < average PPD scores < 0.29) but the pattern of their PPD development did not correspond to a delayed PPD pattern. For example, the cultivar Lelen (cluster 3) displayed a high PPD score at 4 dph (0.41 ± 0.13) with a decrease in PPD score at 7 dph (0.2 ± 0.11). This contradicting result can be partially explained by the difficulty to establish a robust PPD score at 7 dph due to the relatively high microbial contamination observed for the cultivar Lelen. Conversely, cultivar Adira I (cluster 4) displayed low PPD scores at 2 and 4 dph (0.13 ± 0.03 and 0.14 ± 0.01) with a sharp increase at 7 dph (0.5 ± 0.17).

**Fig. 4:**
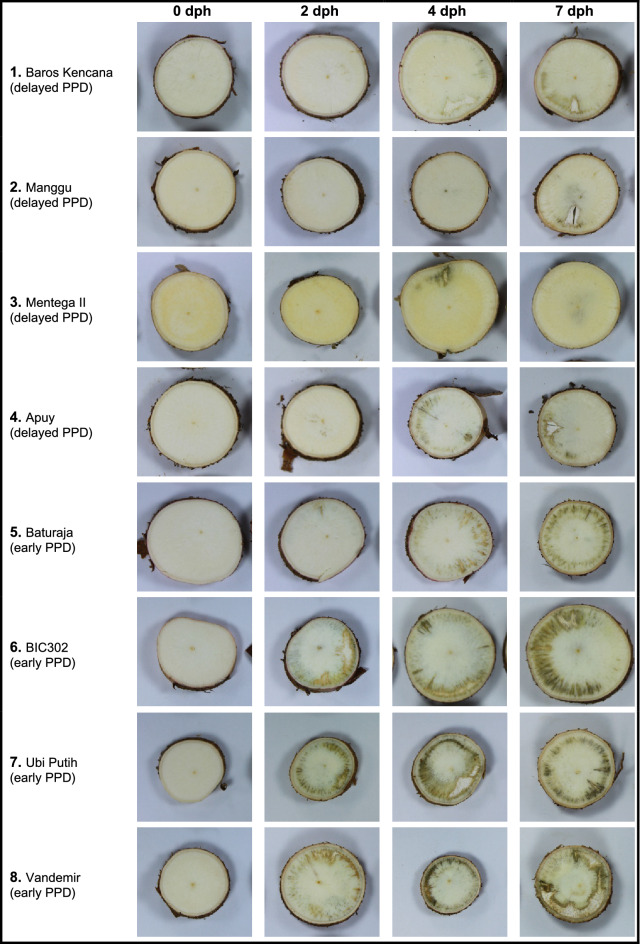
PPD symptoms in selected Indonesian cassava cultivars during the 7 day time course (dph = days post-harvesting)

Based on the percentage of microbial contamination (≤ 11%) and pattern group, we subsequently selected Manggu, MentegaII, Baros Kencana, and Apuy from the clusters 1 and 2 as cultivars with good storage performance and significantly delayed onset of PPD symptoms. The cultivars Baturaja, Vandemir, Ubi Putih, BIC302 from clusters 6 and 7, were selected as early PPD cultivars (Fig. 4).

**Fig. 5:**
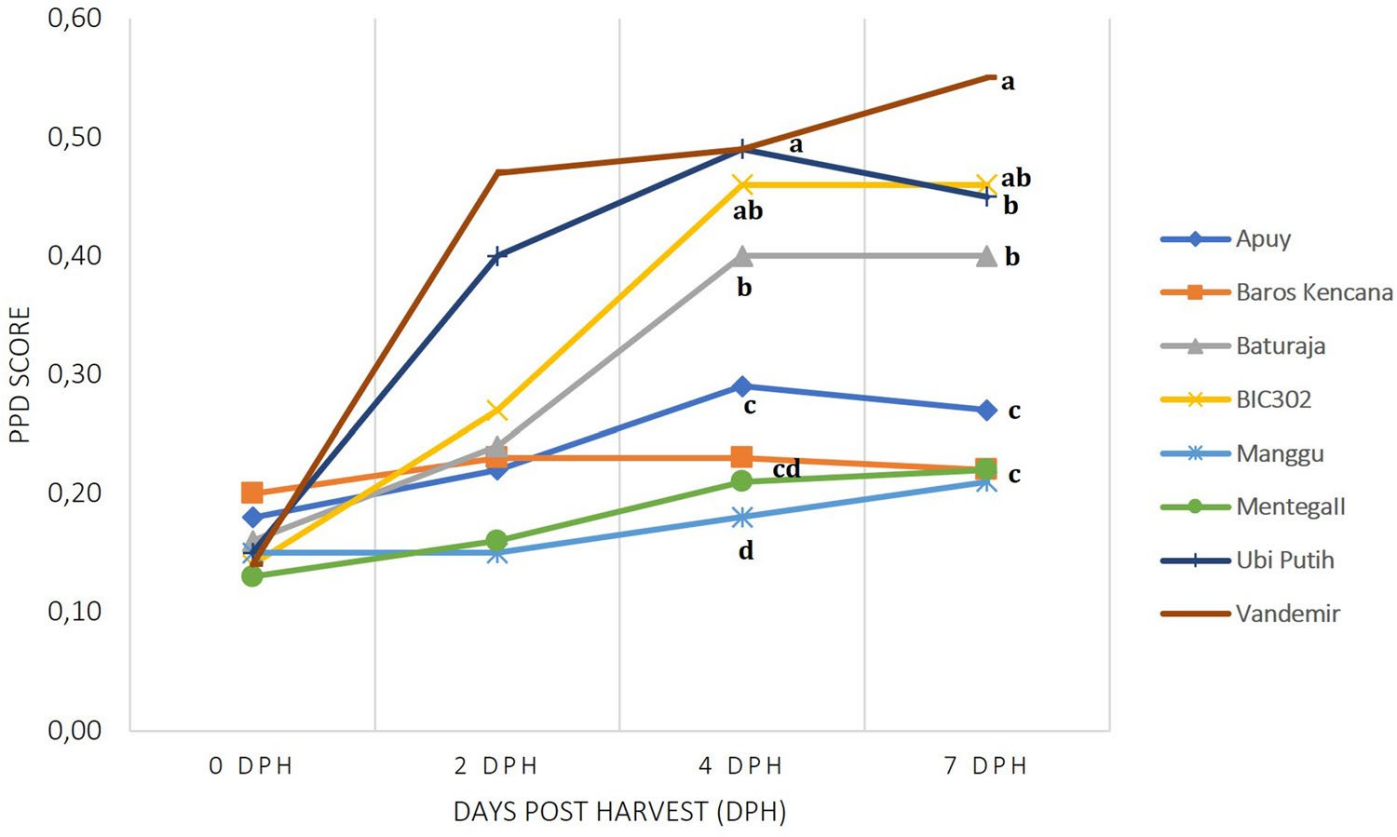
PPD scores of eight selected cassava cultivars. Statistical analysis using one-way ANOVA and LSD post hoc test at each time point (*p* < 0.05)

In order to confirm the robustness of the longitudinal cut method and stability of the PPD trait across seasons, the eight selected cultivars contrasting for PPD onset and development were assessed in a subsequent planting season. Utilizing k-means clustering, the cassava cultivars characterized as “delayed PPD” in the first growing season consistently displayed the same pattern of PPD onset in the second growing season (Additional file 4: Table S5). However, two cassava cultivars (i.e. BIC12 and Ubi Putih) showed delayed PPD onset in the second growing season while they had initially been characterized as early PPD in the first growing season (Additional file 4: Table S5; Additional file 2: Figure S3). The cassava cultivar Baturaja consistently displayed an early PPD phenotype in both growing seasons as confirmed by the ANOVA and LSD post hoc test (Fig. 5, Additional file 2: Figure S3).

**Fig. 6:**
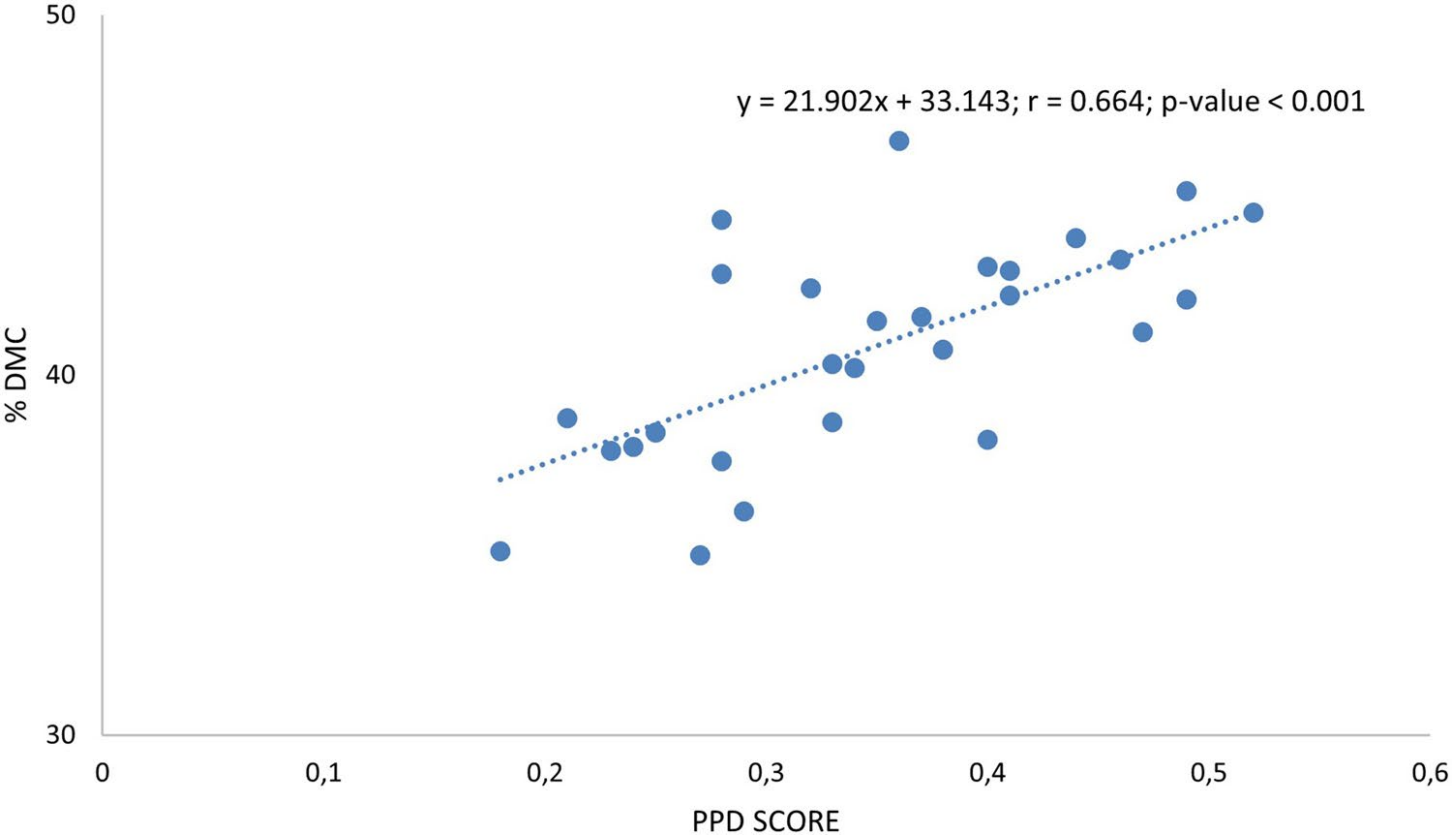
Correlation between PPD score at 4 dph and percentage of dry matter content (%DMC) in 28 cassava cultivars

It should be noted that material from the two growing seasons differed in the growing period as cassava roots were harvested after 8–9 months during the first growing season and after 10–12 months during the second growing season. Despite differences in their growing periods, cassava cultivars had consistent DMC at harvest (Additional file 1: Data S3).

### PPD severity correlates with dry matter content

**Fig. 7:**
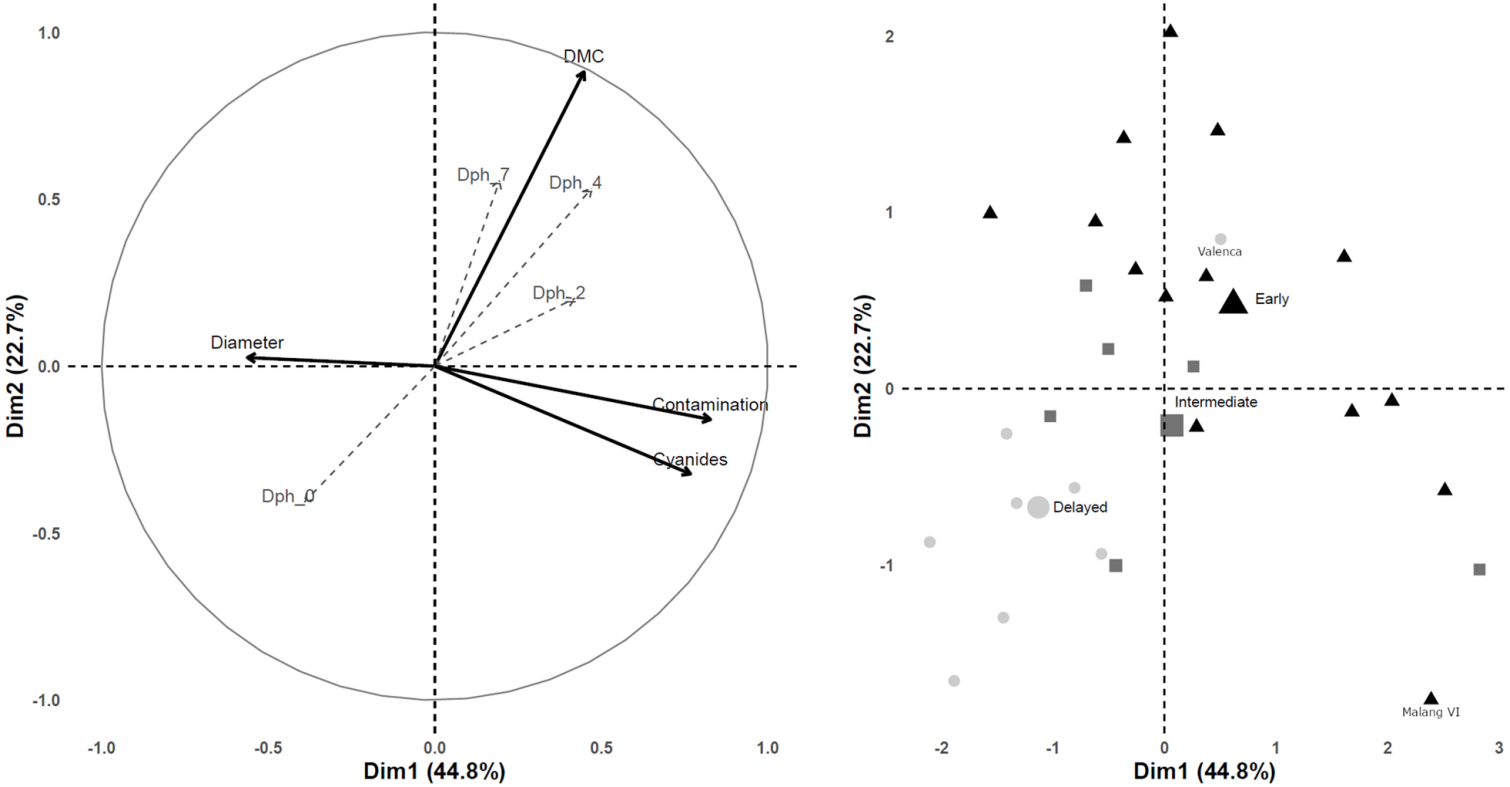
PCA of the twenty-eight cassava cultivars for dimensions 1 and 2. Left: plot of variables, solid arrows represent traits used for computation, dotted arrows (PPD at 0, 2, 4, and 7 dph) represent supplemental traits. Right: Plot of individuals, circle, square and triangle respectively represent delayed, intermediate and early PPD.

At harvest, we measured several cassava agronomic traits, such as yield, length and diameter of the roots, as well as dry matter and cyanogenic potential (Additional file 1: Data S2). Correlations of each trait with PPD score at different time points were determined (Table 3). We observed a significant positive correlation between PPD at 4 and 7 dph and DMC (r = 0.664 and 0.589 respectively, p-value < 0.001) (Fig. 6). This observation also appeared clearly with the PCA (Fig. 7). The positive correlation between PPD scores and DMC of the roots is in agreement with previous studies [10, 17, 37]. Contrasting susceptibility to PPD of all cultivars, except Valenca and Malang VI, could be at least partially explained by the difference in their DMC (Fig. 6).

**Table 3:** Correlation analysis between PPD score at 2 and 4 dph and root length, root diameter, root DMC, and root HCN content

Parameters measured at 0 dph	PPD score (Pearson’s r (df = 26) and p-value)					
	2 dph		4 dph		7 dph	
	r	p-value	r	p-value	r	p-value
length (cm)	0.388	0.041*	0.039	0.842	0.118	0.550
root diameter (cm)	− 0.369	0.053	− 0.354	0.065	− 0.154	0.434
DMC (%)	0.362	0.059	0.664	0.000***	0.589	0.001***
HCN (ppm)	0.290	0.134	0.149	0.450	0.061	0.758

The values show a correlation coefficient (Pearson’s r) between two measured variables

^*^p < 0.05

^***^p < 0.001

Our PPD score data and PCA also revealed a negative correlation but not significant between root diameter and PPD score at 2 and 4 dph (r = − 0.369 and − 0.354 respectively, p-value > 0.05). Additional PPD measurements including other cassava cultivars would be necessary to determine if the PPD symptoms spread faster and more severely in smaller roots. We also observed a significant positive correlation between PPD scores and root lengths at 2 dph (r = 0.388, p-value < 0.05) but this correlation did not appear to be significant at 4 and 7 dph. The positive correlation between PPD scores and root length at 2 dph could indicate that a stronger PPD initiation results from the longer cut, and thereby larger damage and entry point for oxygen. This observation also indicates that roots homogeneous in size should be preferentially used for PPD assessment. Previous studies have reported the use of size parameters to select roots for PPD assessment [10, 12, 22]. The use of cassava roots with a minimum girth of 10 cm has been reported to be associated with low error variation in PPD scores despite the use of visual scoring method [37].

The cyanogenic potential (HCN content) of the cassava roots from the Indonesian cultivars did not correlate with PPD susceptibility. This observation corroborates results from a previous report using the conventional PPD assessment method [10]. Finally, the PCA suggests that cyanogenic potential and microbial contaminations are not correlated with PPD symptoms, but are highly correlated with each other (Additional file 4: Table S6). Both of these traits correlate negatively with the root diameter, suggesting that smaller roots are more likely to have a high cyanogenic potential and are more susceptible to microbial contamination.

## Discussion

In the present study, we established a PPD assessment method based on longitudinal cuts of cassava storage roots and demonstrated its suitability to assess PPD tolerance under an environment conducive to microbial infection of cassava storage roots. The method provided a reliable assessment of PPD until 7 dph with limited microbial contamination. Importantly the method is suitable to initiate a homogenous and rapid induction of PPD symptoms in cassava storage roots. The performance of the newly established method was compared with the standard method using image-based quantification of PPD symptoms. The longitudinal cut method was instrumental to characterize the PPD response in a selection of the most important cassava cultivars in Indonesia. Using a time course evaluation of PPD development the selected cassava cultivars could be classified into the categories of delayed, intermediate, and early PPD.

Some cultivars were identified as delayed and early PPD due to the significantly contrasting PPD scores they received at the early phases and at the completion of the assessment period respectively. On these terms, cassava cultivars of Manggu, MentegaII, Baros Kencana, and Apuy consistently showed consistent resistance to PPD at either early (2 and 4 dph) or late (7 dph) phases of assessment over two growing seasons. Nevertheless, the difficulty was to find the cultivar that consistently showed susceptibility to PPD. Vandemir, Ubi Putih, BIC302, and Baturaja displayed an early onset of PPD in the first growing season. However, Baturaja was the only cultivar which showed a consistent PPD response over the two growing seasons. Our observations confirm the importance of environmental factors in the response to PPD as reported in previous studies [8, 11, 19, 21]. It also highlights the importance of evaluating cassava cultivars for the PPD trait over several years and possibly in different environments [11, 17, 38]. Given its significant and consistent correlation with PPD onset, DMC can also be used as a proxy to estimate the PPD potential of the assessed roots [2].

Measurement of selected root parameters revealed a significant correlation between root length and PPD scores at 2 dph. This observation highlights the importance of selecting cassava storage roots with homogenous sizes to assess PPD tolerance [22, 29, 38]. Indeed, the rapidity and extent of PPD onset could be linked to the level of oxygen entry into the storage roots as it is required to trigger the oxidative burst (reviewed in [33] and [49].

Because of the limitation of model species for the study of storage root physiology [40], the identification of cassava cultivars contrasting for PPD tolerance is of primary interest to decipher the molecular and physiological mechanisms involved in the PPD onset. Previous transcriptomics and proteomics studies [26, 31, 41, 48] using a time course approach on cassava root slices developing PPD symptoms have allowed a preliminary characterization of several pathways deregulated during PPD onset. The use of cassava cultivars contrasting for PPD onset will help to further characterize pathways whose regulation is associated with delayed onset of PPD. The use of the central root section which remains cut-free with the longitudinal cut method is suitable to limit the impact of damage-associated pathways in the characterization of PPD when using root slices for example. The cultivars characterized in the present study could also be used for the introgression of the delayed PPD trait in other farmer-or consumer-preferred cassava cultivars grown in Indonesia. This approach is complementary to the non-destructive sampling procedure that allows identifying biochemical and physiological characteristics of cassava roots immediately at harvest that are associated with delayed PPD [16].

Using the longitudinal cut protocols, we could also confirm the role played by root DMC at harvest on the onset of PPD in cassava storage roots. Our data and previous studies [10, 17, 37] suggest that the correlation between root DMC and PPD susceptibility is cultivar-independent. However, it should be noted that our analysis of 28 cultivars allowed the identification of the cultivar Valenca with high DMC but low PPD incidence. It suggests that the delayed PPD trait is present in various genetic backgrounds and that high DMC and delayed PPD are not mutually exclusive. Given the importance of DMC for industrial processing and applications [18], the use of cultivars such as Valenca could also be prioritized in breeding programmes.

## Supplementary Information

**Additional file 1: Data S1.** Tuber morphology and the origin of 28 Indonesian cassava cultivars. **Data S2.** Measured cassava agronomic traits at harvesting time, including yield, length, diameter of the roots, DMC and HCN contents in 2012. **Data S3.** Harvest time and %DMCs of eight selected cultivars in 2014. The number of biological replicates of PPD assessed roots per cultivar per time point.

**Additional file 2: Figure S1.** Illustrations of fungal contamination in cassava storage roots subjected to PPD assessment method. **Figure S2.** Data analysis of PPD development by utilizing k-means clustering. **Figure S3.** PPD scores of the selected cultivars assessed in 2012 and 2014

**Additional file3: Methods S1.** Schematic diagram of longitudinal cut (LC) methods

**Additional file 4: Table S1.** Temperature and relative humidity in the cassava storing facilities at BRIN (-6.244421823593764, 106.80799907767684). **Table S2.** Preliminary test by using standard PPD assessment method and visual scoring in 2011. **Table S3.** Percentage of the roots affected by microbial contamination during PPD assessment at 2, 4, 7, and 14 dph using longitudinal cut (LC) and standard (S) methods. BR: Biological Replicates (minimum 3 replicates per cultivar per time point). **Table S4.** General Linear Model: microbial contamination versus method; cultivar; and dph. **Table S5.** Data analysis of PPD development of cassava cultivars harvested at two harvesting times by utilizing k-means clustering (Number of classes = 3). **Table S6.** Correlation matrix of root length, root diameter, root DMC, root HCN content, and % contaminated roots at 7 dph. The values show correlation coefficient (Pearson’s r) between two measured variables.

## Data Availability

The authors confirm that the data supporting the findings of this study are available within the article and its supplementary materials.
