# Supplementary material for: A method for rapid and homogenous initiation of post-harvest physiological deterioration in cassava storage roots identifies Indonesian cultivars with improved shelf-life performance
Source: Plant Methods. 2023 Jan 18;19:4. doi: 10.1186/s13007-022-00977-w (PMC9847153; doi:10.1186/s13007-022-00977-w)
Supplement: Supplementary file 1 — Additional file 1: Data S1. Tuber morphology and the origin of 28 Indonesian cassava cultivars. Data S2. Measured cassava agronomic traits at harvesting time, including yield, length, diameter of the roots, DMC and HCN contents in 2012. Data S3. Harvest time and %DMCs of eight selected cultivars in 2014. The number of biological replicates of PPD assessed roots per cultivar per time point. [file 13007_2022_977_MOESM1_ESM.docx]

**Additional file 1: Data S1.** Tuber morphology and the origin of 28 Indonesian cassava cultivars

| **No** | **Cultivars** | **Tuber Morphology** | | | | | | | | **Origin** | | **Biotic Resistance** | **Farmer- / Industry- preferred**  **(consumer acceptability/production application)** | **Variety/ Landrace** |
| --- | --- | --- | --- | --- | --- | --- | --- | --- | --- | --- | --- | --- | --- | --- |
|  |  | **Tuber flesh colour** | **Tuber shape** | **Cortex colour** | **Outer skin colour** | **Cortex thickness** | **Ease of peeling** | **Root constriction** | **Skin texture** |  |  |  |  |  |
|  |  | **1. White**  **2. Cream**  **3. Yellow** | **1. Conical**  **2. Cylinder**  **3. Conical cylinder**  **4. Irregular** | **1. Cream**  **2. Yellow**  **3. Pink**  **4. Purple** | **1. Light brown**  **2. Dark brown**  **3. Cream**  **4. Yellow** | **mm** | **1. Easy**  **2. Difficult** | **3. light green**  **5. dark green**  **7. purple green**  **9. purple** | **3. Smooth**  **5. Intermediate**  **7. Rough** | **City** | **Province** |  |  |  |
| 1 | Adira I | 3 | 2 | 1 | 2 | 1,68 | 1 | 1 | 7 | Bogor | West Java | R, pest: *Tetranichus* sp; R, diseases: *Pseudomonas solacearum*, *Xanthomonas manihotis* | Industry | Variety |
| 2 | Adira IV | NA | NA | NA | NA | NA | NA | NA | NA | Cibinong, Bogor | West Java | Intermediate to pest: *Tetranichus* sp; R, diseases: *Pseudomonas solacearum*, *Xanthomonas manihotis* | Industry | Variety |
| 3 | Apuy | 1 | 2 | 1 | 2 | 1,48 | 1 | 2 | 7 | Cibinong, Bogor | West Java | NA | Farmer | Landrace |
| 4 | Baros Kencana | 1 | 3 | 4 | 2 | 1,92 | 1 | 2 | 5 | Sukabumi | West Java | NA | Farmer | Landrace |
| 5 | Baturaja | 1 | 2 | 4 | 2 | 1,74 | 1 | 2 | 7 | Bogor | West Java | NA | Farmer | Landrace |
| 6 | BIC 302 |  |  |  |  |  |  |  |  | Bogor | West Java | NA | Farmer | Landrace |
| 7 | Darul Hidayah | 1 | 2 | 4 | 1 | 1.23 | 1 | 1 | 7 | Lampung | Lampung (South of Sumatera) | NA | Industry | Variety |
| 8 | Gebang | 1 | 2 | 1 | 2 | 1,7 | 1 | 1 | 5 | Cibinong, Bogor | West Java | NA | Farmer | Landrace |
| 9 | Gempol |  |  |  |  |  |  |  |  | Bogor | West Java | NA | Farmer | Landrace |
| 10 | Kristal Merah | 1 | 2 | 3 | 2 | 2,56 | 1 | 1 | 7 | kalimantan Tengah | Central Kalimantan | NA | Farmer | Landrace |
| 11 | Kristal Putih | 1 | 2 | 1 | 2 | 1,69 | 1 | 1 | 7 | Kalimantan Tengah | Central Kalimantan | NA | Farmer | Landrace |
| 12 | Lelen | 1 | 3 | 2 | 1 | 1,84 | 1 | 1 | 7 | Bantul, Yogyakarta | Special Region of Yogyakarta (Java) | NA | Farmer | Landrace |
| 13 | Lokal Nguneng | 1 | 2 | 1 | 2 | 12:28 | 1 | 1 | 7 | Bogor | West Java | NA | Farmer | Landrace |
| 14 | Malang II | 1 | 3 | 1 | 2 | 1,47 | 1 | 1 | 5 | Malang | East Java | R, pest: *Tetranichus* sp; R, diseases: *Cercospora* sp, Cassava bacterial blight | Industry | Variety |
| 15 | Malang VI | 1 | 1 | 1 | 1 | 1,75 | 1 | 1 | 5 | Malang | East Java | R (Resistant), pest: Tetranichus sp | Industry | Variety |
| 16 | Manggu | 1 | 2 | 1 | 2 | 1,52 | 1 | 1 | 7 | Pasirsuran, Pelabuhan Ratu | West Java | NA | Farmer | Landrace |
| 17 | Mentega I | 3 | 2 | 1 | 2 | 1,52 | 1 | 1 | 7 | Singaparna, Tasikmalaya | West Java | NA | Farmer | Landrace |
| 18 | Mentega II | 3 | 2 | 1 | 1 | 1,43 | 1 | 1 | 5 | Sariwangi, Tasikmalaya | West Java | NA | Farmer | Landrace |
| 19 | Menti | 1 | 2 | 4 | 2 | 1.62 | 1 | 1 | 7 | Karangasem, Wonosari, Jateng | Special Region of Yogyakarta (Java) | NA | Farmer | Landrace |
| 20 | Randu | 1 | 2 | 1 | 2 | 1,37 | 1 | 2 | 7 | Candi Puro, Lumajang | East Java | NA | Farmer | Landrace |
| 21 | Rengganis | 1 | 2 | 1 | 2 | 1,14 | 1 | 1 | 5 | Bogor | West Java | NA | Farmer | Landrace |
| 22 | Roti | 1 | 2 | 1 | 2 | 1,57 | 1 | 1 | 7 | Tanah Sareal, Bogor | West Java | NA | Farmer | Landrace |
| 23 | Sentul | NA | NA | NA | NA | NA | NA | NA | NA | Wuryantoro, Wonogiri | East Java | NA | Farmer | Landrace |
| 24 | Tali | NA | NA | NA | NA | NA | NA | NA | NA | Bogor | West Java | NA | Farmer | Landrace |
| 25 | Ubi Kuning | 3 | 2 | 1 | 2 | 1,64 | 1 | 1 | 7 | Nusa Tenggara Timur | East Nusa Tenggara | NA | Farmer | Landrace |
| 26 | Ubi Putih | 1 | 3 | 1 | 1 | 1,63 | 1 | 1 | 3 | Cerme, Gresik | East Java | NA | Farmer | Landrace |
| 27 | Valenca | 1 | 3 | 4 | 2 | 1,32 | 1 | 2 | 7 | Bogor | West Java | NA | Farmer | Landrace |
| 28 | Vandemir | 1 | 2 | 1 | 2 | 1,36 | 1 | 2 | 7 | Grogol, Kediri | East Java | NA | Farmer | Landrace |

**Additional file 1: Data S2.** Measured cassava agronomic traits at harvesting time, including yield, length, diameter of the roots, DMC and HCN contents in 2012

| **Cultivars** | **Planting Time** | **Harvesting Time** | **# roots** | **Mean root weight (g/plant)** | | | **Mean root length (cm)** | | | **Mean root diameter (cm)** | | | **Mean DMC (%)*** | | | **Total cyanide content (ppm HCN equivalent)**** | | |
| --- | --- | --- | --- | --- | --- | --- | --- | --- | --- | --- | --- | --- | --- | --- | --- | --- | --- | --- |
|  |  |  |  |  |  |  |  |  |  |  |  |  |  |  |  |  |  |  |
| Apuy | 07.09.2011 | 22.05.2012 | 30 | 1789.5 | ± | 1103.0 | 23.2 | ± | 7.9 | 5.2 | ± | 0.7 | 36.2 | ± | 1.4 | 23.6 | ± | 1.3 |
| Baros Kencana |  |  | 30 | 1766.7 | ± | 1064.1 | 27.3 | ± | 12.5 | 5.1 | ± | 1.0 | 37.9 | ± | 1.9 | 49.4 | ± | 8.2 |
| Gebang |  |  | 30 | 1284.8 | ± | 844.7 | 20.2 | ± | 7.5 | 4.8 | ± | 1.2 | 38.0 | ± | 3.5 | 75.5 | ± | 8.7 |
| Menti |  |  | 30 | 3700.0 | ± | 1065.0 | 20.6 | ± | 5.5 | 5.1 | ± | 1.0 | 41.6 | ± | 0.8 | 27.6 | ± | 4.1 |
| Adira I |  | 30.05.2012 | 31 | 1531.3 | ± | 567.7 | 19.3 | ± | 7.8 | 4.7 | ± | 1.0 | 42.8 | ± | 1.3 | 41.6 | ± | 7.9 |
| Adira IV |  |  | 30 | 1488.0 | ± | 844.7 | 21.0 | ± | 6.7 | 5.7 | ± | 1.1 | 35.0 | ± | 2.7 | 61.1 | ± | 8.2 |
| Mentega I |  |  | 30 | 766.7 | ± | 444.2 | 16.9 | ± | 5.0 | 4.1 | ± | 1.0 | 43.8 | ± | 1.1 | 25.2 | ± | 6.7 |
| Mentega II |  |  | 31 | 1894.1 | ± | 1160.0 | 22.0 | ± | 8.2 | 5.1 | ± | 1.1 | 38.8 | ± | 1.1 | 39.7 | ± | 10.0 |
| Roti |  |  | 25 | 744.7 | ± | 403.1 | 17.7 | ± | 7.0 | 4.2 | ± | 1.0 | 38.7 | ± | 1.4 | 24.7 | ± | 9.2 |
| Darul Hidayah |  | 14.06.2012 | 34 | 1107.1 | ± | 859.8 | 25.6 | ± | 8.8 | 4.9 | ± | 0.9 | 37.6 | ± | 4.9 | 56.2 | ± | 15.6 |
| Kristal Merah |  |  | 47 | 1806.7 | ± | 1117.1 | 22.6 | ± | 7.9 | 4.6 | ± | 0.8 | 38.4 | ± | 0.9 | 53.2 | ± | 13.3 |
| Manggu |  |  | 49 | 1854.5 | ± | 1079.2 | 21.4 | ± | 7.1 | 4.6 | ± | 0.7 | 35.1 | ± | 2.1 | 18.9 | ± | 13.8 |
| Ubi Kuning |  |  | 51 | 1282.4 | ± | 767.3 | 25.8 | ± | 8.0 | 3.9 | ± | 0.8 | 40.2 | ± | 3.4 | 30.8 | ± | 21.1 |
| Baturaja | 01.10.2011 | 27.06.2012 | 47 | 1402.9 | ± | 783.7 | 22.5 | ± | 6.6 | 4.5 | ± | 0.9 | 43.0 | ± | 0.3 | 64.0 | ± | 16.9 |
| Malang II |  |  | 27 | 986.7 | ± | 662.6 | 22.1 | ± | 6.6 | 4.7 | ± | 0.9 | 42.4 | ± | 1.4 | 126.9 | ± | 13.3 |
| Malang VI |  |  | 27 | 658.3 | ± | 287.1 | 22.3 | ± | 7.9 | 3.7 | ± | 0.6 | 38.2 | ± | 1.7 | 118.5 | ± | 10.1 |
| Sentul |  |  | 25 | 693.3 | ± | 375.1 | 23.2 | ± | 8.5 | 3.5 | ± | 0.8 | 41.2 | ± | 3.3 | 57.7 | ± | 2.9 |
| Ubi Putih |  |  | 39 | 1194.4 | ± | 379.6 | 21.8 | ± | 5.6 | 4.2 | ± | 0.7 | 42.1 | ± | 0.7 | 44.2 | ± | 3.0 |
| Vandemir |  |  | 36 | 1126.5 | ± | 585.8 | 26.3 | ± | 8.2 | 4.3 | ± | 0.6 | 45.1 | ± | 1.3 | 47.5 | ± | 15.4 |
| BIC302 |  | 05.07.2012 | 28 | 1282.5 | ± | 536.9 | 19.9 | ± | 5.9 | 5.2 | ± | 0.9 | 43.2 | ± | 0.7 | 90.9 | ± | 5.4 |
| Gempol |  |  | 26 | 2084.4 | ± | 1392.3 | 27.2 | ± | 9.1 | 5.4 | ± | 1.0 | 42.2 | ± | 1.4 | 24.7 | ± | 5.4 |
| Kristal Putih |  |  | 23 | 957.1 | ± | 450.2 | 23.8 | ± | 8.0 | 4.3 | ± | 0.6 | 40.7 | ± | 5.4 | 83.7 | ± | 13.5 |
| Lelen |  |  | 36 | 1336.8 | ± | 628.2 | 19.6 | ± | 6.3 | 4.9 | ± | 0.8 | 42.9 | ± | 1.1 | 89.0 | ± | 21.8 |
| Rengganis |  |  | 21 | 1065.0 | ± | 470.3 | 16.9 | ± | 7.1 | 5.1 | ± | 0.9 | 46.5 | ± | 2.7 | 32.5 | ± | 13.1 |
| Valenca |  |  | 31 | 1087.5 | ± | 488.7 | 22.3 | ± | 8.2 | 4.7 | ± | 1.0 | 44.3 | ± | 0.4 | 89.9 | ± | 3.9 |
| Lokal Nguneng |  | 10.07.2012 | 19 | 1060.0 | ± | 814.2 | 21.3 | ± | 9.4 | 3.7 | ± | 0.8 | 40.3 | ± | 4.7 | 96.4 | ± | 15.8 |
| Randu |  |  | 26 | 1096.2 | ± | 488.0 | 16.9 | ± | 5.4 | 5.2 | ± | 0.8 | 41.5 | ± | 2.7 | 50.8 | ± | 13.4 |
| Tali |  |  | 26 | 2085.7 | ± | 1086.2 | 24.9 | ± | 12.3 | 4.5 | ± | 0.8 | 44.5 | ± | 1.0 | 74.7 | ± | 4.4 |
|  |  | **Remarks:** | *n=4 | |  | **n=3 | | | |  |  |  |  |  |  |  |  |  |
|  |  |  |  | |  |  | | | |  |  |  |  |  |  |  |  |  |

**Additional file 1: Data S3.** Harvest time and %DMCs of eight selected cultivars in 2014.

| **Cultivars** | **PPD status** | **Planting Time** | **Harvesting Time** | **Harvest Time (weeks after planting)** | **%DMC (2012)** | | | **%DMC (2014)** | | |
| --- | --- | --- | --- | --- | --- | --- | --- | --- | --- | --- |
| Apuy | Delayed | 13.05.2013 | 27.04.2014 | 46 | 36.2 | ± | 1.4 | 40.10 | ± | 3.26 |
| Baros Kencana | Delayed | 15.04.2013 | 22.04.2014 | 47 | 37.9 | ± | 1.9 | 45.04 | ± | 5.66 |
| Manggu | Delayed | 24.04.2013 | 22.04.2014 | 48 | 35.1 | ± | 2.1 | 42.45 | ± | 4.42 |
| Mentega II | Delayed | 18.04.2013 | 22.04.2014 | 48 | 38.8 | ± | 1.1 | 35.98 | ± | 6.12 |
| Baturaja | Early | 08.07.2013 | 27.04.2014 | 39 | 43.0 | ± | 0.3 | 42.68 | ± | 2.72 |
| BIC 302 | Early | 24.05.2013 | 22.04.2014 | 44 | 43.2 | ± | 0.7 | 36.43 | ± | 2.72 |
| Ubi Putih | Early | 24.05.2013 | 22.04.2014 | 44 | 42.1 | ± | 0.7 | 42.52 | ± | 3.23 |
| Vandemir | Early | 24.05.2013 | 22.04.2014 | 44 | 45.1 | ± | 1.3 | 40.57 | ± | 2.06 |

**Additional file 1: Data S4.** The number of biological replicates of PPD assessed roots per cultivar per time point.

| **Cultivars** | **Biological Replicates** | | | | **Total** | **Cultivars** | **Biological Replicates** | | | | **Total** |
| --- | --- | --- | --- | --- | --- | --- | --- | --- | --- | --- | --- |
|  | **0 dph** | **2 dph** | **4 dph** | **7 dph** |  |  | **0 dph** | **2 dph** | **4 dph** | **7 dph** |  |
| Adira I | 5 | 6 | 6 | 6 | 23 | Malang II | 6 | 5 | 6 | 3 | 20 |
| Adira IV | 6 | 6 | 6 | 6 | 24 | Malang VI | 6 | 5 | 6 | 5 | 22 |
| Apuy | 6 | 5 | 6 | 5 | 22 | Menti | 6 | 6 | 6 | 5 | 23 |
| BIC | 6 | 6 | 6 | 4 | 22 | Mentega I | 6 | 6 | 6 | 6 | 24 |
| Baros Kencana | 6 | 6 | 6 | 6 | 24 | Mentega II | 5 | 6 | 6 | 6 | 23 |
| Baturaja | 9 | 10 | 10 | 10 | 39 | Rengganis | 3 | 4 | 4 | 4 | 15 |
| Darul Hidayah | 7 | 7 | 6 | 5 | 25 | Randu | 5 | 5 | 6 | 4 | 20 |
| Gebang | 6 | 5 | 6 | 5 | 22 | Roti | 4 | 5 | 5 | 5 | 19 |
| Gempol | 6 | 6 | 5 | 4 | 21 | Sentul | 5 | 4 | 6 | 4 | 19 |
| Kristal Merah | 10 | 10 | 10 | 10 | 40 | Tali | 5 | 5 | 4 | 6 | 20 |
| Kristal Putih | 4 | 4 | 5 | 3 | 16 | Ubi Kuning | 9 | 10 | 10 | 9 | 38 |
| Lelen | 7 | 8 | 6 | 3 | 24 | Ubi Putih | 9 | 8 | 9 | 7 | 33 |
| Lokal Nguneng | 4 | 4 | 4 | 2 | 14 | Vandemir | 3 | 7 | 9 | 8 | 27 |
| Manggu | 8 | 9 | 8 | 8 | 33 | Valenca | 5 | 6 | 6 | 6 | 23 |
