# Supplementary material for: A method for rapid and homogenous initiation of post-harvest physiological deterioration in cassava storage roots identifies Indonesian cultivars with improved shelf-life performance
Source: Plant Methods. 2023 Jan 18;19:4. doi: 10.1186/s13007-022-00977-w (PMC9847153; doi:10.1186/s13007-022-00977-w)
Supplement: Supplementary file 2 — Additional file 2: Figure S1. Illustrations of fungal contamination in cassava storage roots subjected to PPD assessment method. Figure S2. Data analysis of PPD development by utilizing k-means clustering. Figure S3. PPD scores of the selected cultivars assessed in 2012 and 2014 [file 13007_2022_977_MOESM2_ESM.docx]

|  | **Bacterial Contaminated**  **i.e. Darul Hidayah_longitudinal cut method_7dph** | **Fungal Contaminated**  **i.e. Apuy_standard method_14dph** | **PPD**  **i.e. Baturaja_longitudinal cut method_14dph** |
| --- | --- | --- | --- |
| RGB color | 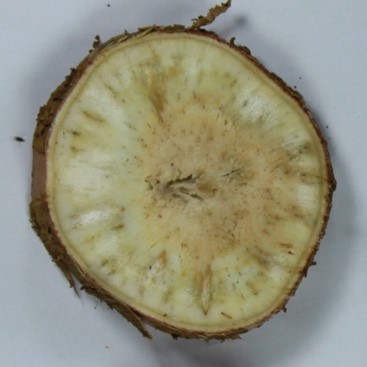 | 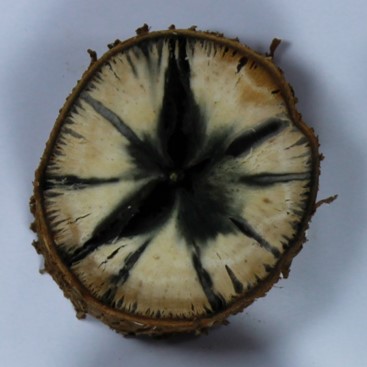 | 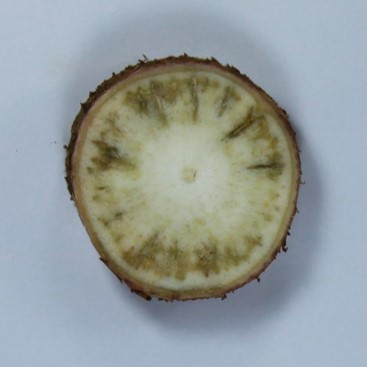 |
| Grayscale color | 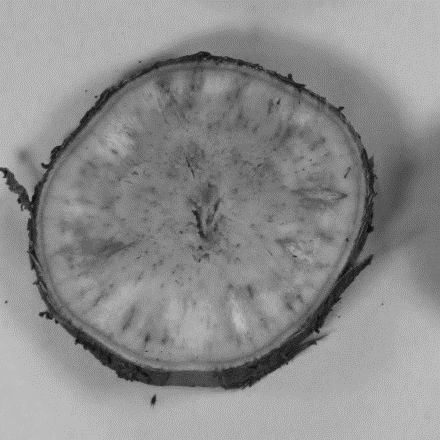 | 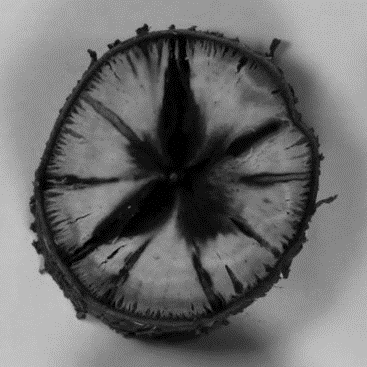 | 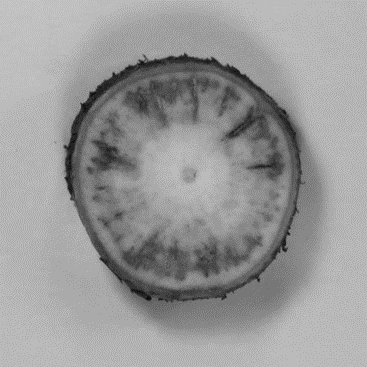 |
|  |  |  |  |

**Additional file 2: Figure S1**. Illustrations of fungal contamination in cassava storage roots subjected to PPD assessment method.


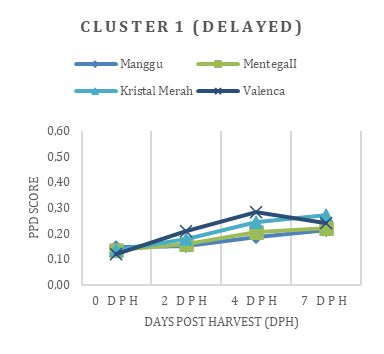

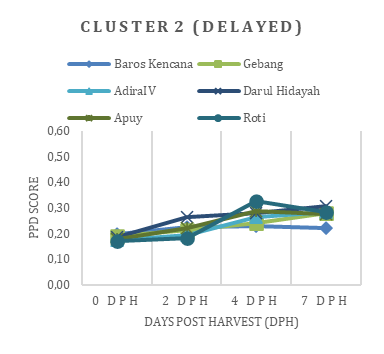

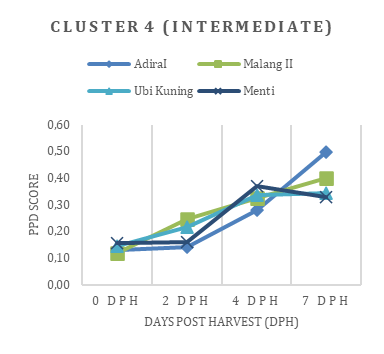

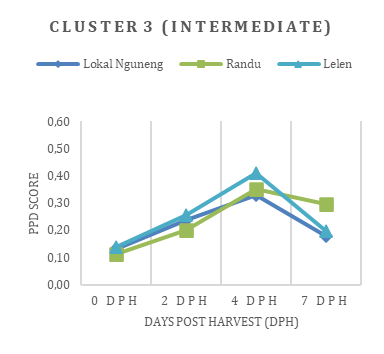

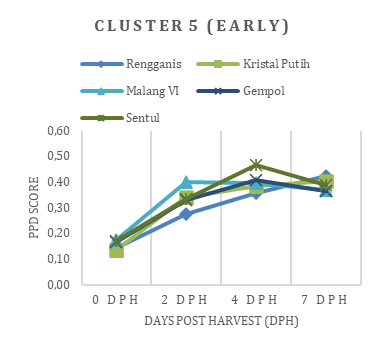

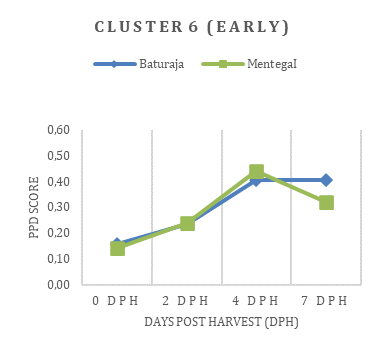

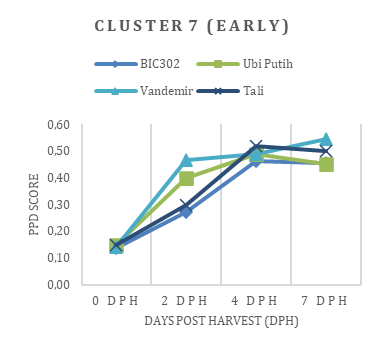


**Additional file 2: Figure S2.** Data analysis of PPD development by utilizing k-means clustering.

c

bc

**Additional file 2: Figure S3.** PPD scores of the selected cultivars assessed in 2012 and 2014.
