# Supplementary material for: A method for rapid and homogenous initiation of post-harvest physiological deterioration in cassava storage roots identifies Indonesian cultivars with improved shelf-life performance
Source: Plant Methods. 2023 Jan 18;19:4. doi: 10.1186/s13007-022-00977-w (PMC9847153; doi:10.1186/s13007-022-00977-w)
Supplement: Supplementary file 3 — Additional file3: Methods S1. Schematic diagram of longitudinal cut (LC) methods [file 13007_2022_977_MOESM3_ESM.docx]

**PPD Assessment Methods: Longitudinal Cut**

Description of the Illustration in Figure 1

PPD Score

**Additional file 3: Methods S1.** Schematic diagram of longitudinal cut (LC) methods.

1. **Samples collection at harvesting time**

Storage roots are harvested from several cassava plants to obtain sufficient replications number for PPD assessment at each time point (e.g., minimum of 24 roots for four-time points including control measured at harvesting time). To minimize the error variation, cassava roots with homogenous sizes are preferentially used.

1. **Longitudinal cut**

The longitudinal cut is performed with a paper cutter by giving a shallow cut (3 mm-deep cuts) at both ends of the roots and leaving the 10,5 cm long central part of the root undamaged. Before each treatment, paper cutter was previously sterilised by being dipped in ethanol solution and sterile distillated water to reduce microbial occurrences.

1. **Samples storage**

Following the longitudinal cut, all treated cassava storage roots except control are stored at a storage place that is protected from direct sun, rain, and rodents, but exposed to air. To have an effective induction for PPD symptoms, the temperature and relative humidity of the storage place are adjusted to the environmental condition.

1. **PPD assessment at each time point**

Some treated roots replicates (e.g., minimum 6 roots) are assessed for their PPD as control at harvesting time. The PPD assessment includes the slicing of the undamaged central part of the root into 7 transversal slices (ca. 1,5 cm thickness) and arranging from proximal to distal ends on the A4 size paper for being photographed. The photo images for all samples are taken in the same condition by using a camera stand and a dark room. The other root replicates were assessed at the respective time points by the same methods as that for the control.

1. **Matlab-based image processing tool**

Images of proximal, central and distal (A1, A2, and A3) slices are used for PPD score analysis using a previously established image-based PPD scoring procedure (Vanderschuren et al., 2014). PPD score was defined from the following formula:

PPD score = (Quantile 97.5% - Quantile 2.5%)

Quantile 97.5%

PPD score is the average of the PPD scores from the proximal, central, and distal slices.
