# Supplementary material for: A method for rapid and homogenous initiation of post-harvest physiological deterioration in cassava storage roots identifies Indonesian cultivars with improved shelf-life performance
Source: Plant Methods. 2023 Jan 18;19:4. doi: 10.1186/s13007-022-00977-w (PMC9847153; doi:10.1186/s13007-022-00977-w)
Supplement: Supplementary file 4 — Additional file 4: Table S1. Temperature and relative humidity in the cassava storing facilities at BRIN (-6.244421823593764, 106.80799907767684). Table S2. Preliminary test by using standard PPD assessment method and visual scoring in 2011. Table S3. Percentage of the roots affected by microbial contamination during PPD assessment at 2, 4, 7, and 14 dph using longitudinal cut (LC) and standard (S) methods. BR: Biological Replicates (minimum 3 replicates per cultivar per time point). Table S4. General Linear Model: microbial contamination versus method; cultivar; and dph. Table S5. Data analysis of PPD development of cassava cultivars harvested at two harvesting times by utilizing k-means clustering (Number of classes = 3). Table S6. Correlation matrix of root length, root diameter, root DMC, root HCN content, and % contaminated roots at 7 dph. The values show correlation coefficient (Pearson’s r) between two measured variables. [file 13007_2022_977_MOESM4_ESM.docx]

**Supplementary Table 1.** Temperature and relative humidity in the cassava storing facilities at BRIN (-6.244421823593764, 106.80799907767684)

| **Date** | **8:00** | | **12:00** | | **16:00** | |
| --- | --- | --- | --- | --- | --- | --- |
|  | **T(^o^C)** | **RH (%)** | **T(^o^C)** | **RH (%)** | **T(^o^C)** | **RH (%)** |
| **May-July 2012** | 24.68 ± 1.60 | 77.35 ± 10.86 | 32.12 ± 1.31 | 48.86 ± 8.94 | 28.48 ± 1.57 | 60.31 ± 10.30 |

**Supplementary Table 2.** Preliminary test by using standard PPD assessment method and visual scoring in 2011

| **No** | **Cultivars** | **PPD Score (by visual scoring)** | | | | | | | | | | | |
| --- | --- | --- | --- | --- | --- | --- | --- | --- | --- | --- | --- | --- | --- |
|  |  | **2 dph** | | | **4 dph** | | | **7 dph** | | | **14 dph** | | |
| 1 | Adira I | 0,0 | ± | 0,00 | 5,1 | ± | 6,07 | 8,98 | ± | 8,42 | N/A* | | |
| 2 | Adira IV | 0,0 | ± | 0,00 | 1,0 | ± | 0,84 | 5,27 | ± | 7,28 | N/A* | | |
| 3 | Mentega I | 0,4 | ± | 0,45 | 2,7 | ± | 2,57 | 24,76 | ± | 23,93 | 44,76 | ± | 50,13 |
| 4 | Mentega II | 4,3 | ± | 3,35 | 6,9 | ± | 1,26 | 39,66 | ± | 42,04 | 36,90 | ± | 10,01 |
| 5 | Roti | 0,0 | ± | 0,00 | 3,7 | ± | 2,41 | 31,09 | ± | 30,55 | N/A* | | |

Remarks: * the roots with N/A of biological replicates were due to microbial deterioration

**Supplementary Table 3.** Percentage of the roots affected by microbial contamination during PPD assessment at 2, 4, 7, and 14 dph using longitudinal cut (LC) and standard (S) methods. BR: Biological Replicates (minimum 3 replicates per cultivar per time point)

| **No** | **Cultivars** | **2 dph** | | **4 dph** | | **7 dph** | | | | **14 dph** | | | |
| --- | --- | --- | --- | --- | --- | --- | --- | --- | --- | --- | --- | --- | --- |
|  |  | **LC** | **S** | **LC** | **S** | **BR** | **LC** | **BR** | **S** | **BR** | **LC** | **BR** | **S** |
| 1 | Adira I | 0% | 0% | 0% | 0% | 6 | 0% | 6 | 33% | 5 | 0% | N/A* | 100% |
| 2 | Adira IV | 0% | 0% | 0% | 0% | 6 | 0% | 6 | 33% | 5 | 40% | 4 | 75% |
| 3 | Apuy | 0% | 0% | 0% | 0% | 5 | 0% | 6 | 0% | 5 | 60% | 6 | 100% |
| 4 | Baros Kencana | 0% | 0% | 0% | 0% | 6 | 0% | 5 | 40% | 6 | 33% | 7 | 100% |
| 5 | Gebang | 0% | 0% | 0% | 0% | 5 | 0% | 5 | 60% | 6 | 67% | 6 | 67% |
| 6 | Mentega I | 0% | 0% | 0% | 0% | 6 | 0% | 6 | 33% | 4 | 25% | 2* | 50% |
| 7 | Mentega II | 0% | 0% | 0% | 0% | 6 | 0% | 6 | 17% | 6 | 67% | 4 | 25% |
| 8 | Menti | 0% | 0% | 0% | 0% | 6 | 17% | 6 | 17% | 6 | 67% | 6 | 100% |
| 9 | Roti | 0% | 0% | 0% | 0% | 5 | 0% | 5 | 0% | 5 | 0% | 2* | 100% |

Remarks: * the roots with less number or N/A of biological replicates were due to microbial deterioration

**Supplementary Table 4.** General Linear Model: microbial contamination versus method; cultivar; and dph.

**Method**

| Factor coding | (-1; 0; +1) |
| --- | --- |

**Factor Information**

| **Factor** | **Type** | **Levels** | **Values** |
| --- | --- | --- | --- |
| method | Fixed | 2 | Longitudinal Cut (LC); Standard Assessment Method (S) |
| cultivar | Fixed | 9 | AdiraI; AdiraIV; Apuy; Baros Kencana; Gebang; MentegaI; MentegaII; Menti; Roti |
| dph | Fixed | 2 | 7; 14 |

**Analysis of Variance**

| **Source** | **DF** | **Adj SS** | **Adj MS** | **F-Value** | **P-Value** |
| --- | --- | --- | --- | --- | --- |
| method | 1 | 3,6404 | 3,6404 | 26,71 | 0,000 |
| cultivar | 8 | 1,3545 | 0,1693 | 1,24 | 0,278 |
| dph | 1 | 8,4450 | 8,4450 | 61,96 | 0,000 |
| Method*Cultivar | 8 | 2,8192 | 0,3524 | 2,59 | 0,011 |
| Method*dph | 1 | 0,3800 | 0,3800 | 2,79 | 0,097 |
| Cultivar*dph | 8 | 1,2537 | 0,1567 | 1,15 | 0,333 |
| Method*Cultivar*dph | 8 | 2,0398 | 0,2550 | 1,87 | 0,068 |
| Error | 152 | 20,7167 | 0,1363 |  |  |
| Total | 187 | 42,5266 |  |  |  |

**Model Summary**

| **S** | **R-sq** | **R-sq(adj)** | **R-sq(pred)** |
| --- | --- | --- | --- |
| 0,369180 | 51,29% | 40,07% | * |

**Coefficients**

| **Term** | **Coef** | **SE Coef** | **T-Value** | **P-Value** | **VIF** |
| --- | --- | --- | --- | --- | --- |
| Constant | 0,3681 | 0,0291 | 12,64 | 0,000 |  |
| Method |  |  |  |  |  |
| LC | -0,1505 | 0,0291 | -5,17 | 0,000 | 1,17 |
| Cultivar |  |  |  |  |  |
| AdiraI | -0,035 | 0,105 | -0,33 | 0,741 | 2,83 |
| AdiraIV | 0,0028 | 0,0777 | 0,04 | 0,972 | 1,68 |
| Apuy | 0,0319 | 0,0755 | 0,42 | 0,673 | 1,63 |
| Baros Kencana | 0,0653 | 0,0730 | 0,89 | 0,373 | 1,59 |
| Gebang | 0,1153 | 0,0755 | 1,53 | 0,129 | 1,63 |
| MentegaI | -0,0972 | 0,0896 | -1,09 | 0,280 | 2,06 |
| MentegaII | -0,0972 | 0,0763 | -1,27 | 0,204 | 1,66 |
| Menti | 0,1319 | 0,0726 | 1,82 | 0,071 | 1,57 |
| dph |  |  |  |  |  |
| 7 | -0,2292 | 0,0291 | -7,87 | 0,000 | 1,16 |
| Method*Cultivar |  |  |  |  |  |
| LC AdiraI | -0,183 | 0,105 | -1,74 | 0,083 | 2,83 |
| LC AdiraIV | -0,0204 | 0,0777 | -0,26 | 0,794 | 1,68 |
| LC Apuy | 0,0505 | 0,0755 | 0,67 | 0,505 | 1,63 |
| LC Baros Kencana | -0,1162 | 0,0730 | -1,59 | 0,113 | 1,60 |
| LC Gebang | 0,0005 | 0,0755 | 0,01 | 0,995 | 1,63 |
| LC MentegaI | 0,0046 | 0,0896 | 0,05 | 0,959 | 2,06 |
| LC MentegaII | 0,2963 | 0,0763 | 3,88 | 0,000 | 1,66 |
| LC Menti | 0,0671 | 0,0726 | 0,93 | 0,356 | 1,58 |
| Method*dph |  |  |  |  |  |
| LC 7 | 0,0486 | 0,0291 | 1,67 | 0,097 | 1,17 |
| Cultivar*dph |  |  |  |  |  |
| AdiraI 7 | 0,062 | 0,105 | 0,60 | 0,552 | 2,82 |
| AdiraIV 7 | 0,0250 | 0,0777 | 0,32 | 0,748 | 1,68 |
| Apuy 7 | -0,1708 | 0,0755 | -2,26 | 0,025 | 1,63 |
| Baros Kencana 7 | -0,0042 | 0,0730 | -0,06 | 0,955 | 1,60 |
| Gebang 7 | 0,0458 | 0,0755 | 0,61 | 0,545 | 1,63 |
| MentegaI 7 | 0,1250 | 0,0896 | 1,40 | 0,165 | 2,06 |
| MentegaII 7 | 0,0417 | 0,0763 | 0,55 | 0,586 | 1,66 |
| Menti 7 | -0,1042 | 0,0726 | -1,44 | 0,153 | 1,58 |
| Method*Cultivar*dph |  |  |  |  |  |
| LC AdiraI 7 | 0,118 | 0,105 | 1,13 | 0,262 | 2,83 |
| LC AdiraIV 7 | -0,0444 | 0,0777 | -0,57 | 0,568 | 1,68 |
| LC Apuy 7 | 0,0514 | 0,0755 | 0,68 | 0,497 | 1,63 |
| LC Baros Kencana 7 | 0,0181 | 0,0730 | 0,25 | 0,805 | 1,60 |
| LC Gebang 7 | -0,1986 | 0,0755 | -2,63 | 0,009 | 1,63 |
| LC MentegaI 7 | -0,0694 | 0,0896 | -0,78 | 0,439 | 2,06 |
| LC MentegaII 7 | -0,1111 | 0,0763 | -1,46 | 0,147 | 1,66 |
| LC Menti 7 | 0,0347 | 0,0726 | 0,48 | 0,633 | 1,58 |

**Supplementary Table 5.** Data analysis of PPD development of cassava cultivars harvested at two harvesting times by utilizing k-means clustering (Number of classes = 3).

| Class | 1 | 2 | 3 |
| --- | --- | --- | --- |
| Objects | 10 | 3 | 2 |
| Sum of weights | 10 | 3 | 2 |
| Within-class variance | 0.004 | 0.011 | 0.007 |
| Minimum distance to centroid | 0.024 | 0.017 | 0.060 |
| Average distance to centroid | 0.056 | 0.076 | 0.060 |
| Maximum distance to centroid | 0.107 | 0.111 | 0.060 |
|  | Apuy_2012 | BIC12_2012 | Ubi Putih_2012 |
|  | Apuy_2014 | Baturaja_2012 | Vandemir_2012 |
|  | BIC12_2014 | Baturaja_2014 |  |
|  | Baros Kencana_2012 |  |  |
|  | Baros Kencana_2014 |  |  |
|  | Manggu_2012 |  |  |
|  | Manggu_2014 |  |  |
|  | Mentega II_2012 |  |  |
|  | Mentega II_2014 |  |  |
|  | Ubi Putih_2014 |  |  |
|  |  |  |  |

| ***Parameters measured at 0 dph*** | ***Length (cm)*** | | ***Diameter (mm)*** | | ***DMC (%)*** | | ***HCN (ppm)*** | | ***%contaminated  roots at 7 dph*** | |
| --- | --- | --- | --- | --- | --- | --- | --- | --- | --- | --- |
|  | ***r*** | ***p-value*** | ***r*** | ***p-value*** | ***r*** | ***p-value*** | ***r*** | ***p-value*** | ***r*** | ***p-value*** |
| Length (cm) | 1 |  |  |  |  |  |  |  |  |  |
| Diameter (mm) | -0,059 | 0,766 | 1 |  |  |  |  |  |  |  |
| DMC (%) | -0,057 | 0,773 | -0,086 | 0,664 | 1 |  |  |  |  |  |
| HCN (ppm) | 0,155 | 0,432 | -0,192 | 0,329 | 0,133 | 0,500 | 1 |  |  |  |
| Percentage of contaminated roots at 7 dph | -0,152 | 0,439 | -0,268 | 0,167 | 0,215 | 0,271 | 0,523 | **0,004**** | 1 |  |

**Supplementary Table 6.** Correlation matrix of root length, root diameter, root DMC, root HCN content, and % contaminated roots at 7 dph. The values show correlation coefficient (Pearson’s r) between two measured variables.

Significant codes: p < 0.01 (**)
